# Differences and Similarities in the Clinicopathological Features of Pancreatic Neuroendocrine Tumors in China and the United States: A Multicenter Study

**DOI:** 10.1097/MD.0000000000002836

**Published:** 2016-02-18

**Authors:** Li-Ming Zhu, Laura Tang, Xin-Wei Qiao, Edward Wolin, Nicholas N. Nissen, Deepti Dhall, Jie Chen, Lin Shen, Yihebali Chi, Yao-Zong Yuan, Qi-Wen Ben, Bin Lv, Ya-Ru Zhou, Chun-Mei Bai, Jie Chen, Yu-Li Song, Tian-Tian Song, Chong-Mei Lu, Run Yu, Yuan-Jia Chen

**Affiliations:** From the Department of Gastroenterology, Peking Union Medical College Hospital, Peking Union Medical College, Chinese Academy of Medical Sciences, Beijing (L-MZ, X-WQ, Y-LS, T-TS, C-ML, Y-JC); Department of Pathology, Memorial Sloan-Kettering Cancer Center, New York, NY (LT); Markey Cancer Center, University of Kentucky, Lexington, KY (EW); Department of Surgery (NNN); Department of Pathology, Cedars-Sinai Medical Center, Los Angeles, CA (DD); Department of Gastroenterology, the First Affiliated Hospital, Sun Yat-Sen University, Guangzhou (JC); Department of Gastrointestinal Medical Oncology, Peking University School of Oncology, Beijing Cancer Hospital and Institute (LS); Department of Medical Oncology, Cancer Hospital, Chinese Academy of Medical Sciences, Peking Union Medical College, Beijing (YC); Department of Gastroenterology, Ruijin Hospital, Shanghai Jiaotong University, Shanghai (Y-ZY, Q-WB); Department of Gastroenterology, the First Affiliated Hospital of Zhejiang Chinese Medical University, Hangzhou (BL); Department of Endocrinology, the Third Hospital of Hebei Medical University, Shijiazhuang (Y-RZ); Department of Oncology (C-MB); Department of Pathology, Peking Union Medical College Hospital, Peking Union Medical College, Chinese Academy of Medical Sciences, Beijing (JC); and Division of Endocrinology and Carcinoid and Neuroendocrine Tumor Center, Cedars-Sinai Medical Center, University of California Los Angeles, Los Angeles, CA (RY).

## Abstract

The presentation, pathology, and prognosis of pancreatic neuroendocrine tumors (PNETs) in Asian patients have not been studied in large cohorts. We hypothesized that the clinicopathological features of PNETs of Chinese patients might be different from those of US patients. The objectives of this study were to address whether PNETs in Chinese patients exhibit unique clinicopathological features and natural history, and can be graded and staged using the WHO/ENETS criteria.

This is a retrospective review of medical records of patients with PNETs in multiple academic medical centers in China (7) and the United States (2). Tumor grading and staging were based on WHO/ENETS criteria. The clinicopathological features of PNETs of Chinese and US patients were compared. Univariate and multivariate analyses were performed to find associations between survival and patient demographics, tumor grade and stage, and other clinicopathological characteristics.

A total of 977 (527 Chinese and 450 US) patients with PNETs were studied. In general, Chinese patients were younger than US patients (median age 46 vs 56 years). In Chinese patients, insulinomas were the most common (52.2%), followed by nonfunctional tumors (39.7%), whereas the order was reversed in US patients. Tumor grade distribution was similar in the 2 countries (G1: 57.5% vs 55.0%; G2: 38.5% vs 41.3%; and G3: 4.0% vs 3.7%). However, age, primary tumor size, primary tumor location, grade, and stage of subtypes of PNETs were significantly different between the 2 countries. The Chinese nonfunctional tumors were significantly larger than US ones (median size 4 vs 3 cm) and more frequently located in the head/neck region (54.9% vs 34.8%). The Chinese and US insulinomas were similar in size (median 1.5 cm) but the Chinese insulinomas relatively more frequently located in the head/neck region (48.3% vs 26.1%). Higher grade, advanced stage, metastasis, and larger primary tumor size were significantly associated with unfavorable survival in both countries.

Several clinicopathological differences are found between Chinese and US PNETs but the PNETs of both countries follow a similar natural history. The WHO tumor grading and ENETS staging criteria are applicable to both Chinese and US patients.

## Abstract 2

Supplemental Digital Content is available in the text

## INTRODUCTION

Pancreatic neuroendocrine tumors (PNETs) are a group of uncommon tumors arising in the endocrine pancreas but the incidence of these tumors has increased worldwide.^1–6^ They are classified into subgroups accordingly to various criteria.^1,2,5^ Based on whether they cause clinical hormonal excess syndromes, the PNETs are classified as functional or nonfunctional. Using proliferative markers, PNETs are categorized as low-, intermediate-, or high-grade.^7–9^ Using the TNM (tumor, node, and metastasis) system, PNETs are divided into 4 stages of tumor advancement.^8–10^ PNETs grade and stage are the major determinants of prognosis.^10^

The above tumor taxonomy has largely been based on PNETs in European and US patients. PNETs are well known in Asian countries although the epidemiology is not clearly characterized.^6,11–16^ There have not been large studies of PNETs in Asian patients. The presentation, pathology, and prognosis of PNETs in Asian patients are not clearly described due to the limited number of patients in existing studies. It is not known whether PNETs in Asian patients exhibit unique natural history or can be graded and staged using available criteria.

The most populous and third largest country, China probably has the greatest PNET burden per country.^11–14^ We thus retrospectively compared the presentation, pathology, and prognosis of PNETs in Chinese and US patients in a multicenter study.

## METHODS

### Data Retrieval

A total of 977 patients with histological diagnosis of PNETs from 1983 to 2012 at each participating hospital were studied; these included 344 patients from Peking Union Medical College Hospital, 32 from Cancer Institute and Hospital of Chinese Academy of Medical Sciences, 24 from Beijing Cancer Hospital, all in Beijing, 7 from the Third Hospital of Hebei Medical University, Shijiazhuang, Hebei Province, all in the north of China; 60 from the First Affiliated Hospital of Sun Yat-Sen University, Guangzhou, Guangdong Province, 54 from Ruijin Hospital of Shanghai Jiao Tong University, Shanghai, 6 from the First Affiliated Hospital of Zhejiang Chinese Medical University, Hangzhou, Zhejiang Province, all in the south of China; 342 from Memorial Sloan Kettering Cancer Center, New York, and 108 from Cedars-Sinai Medical Center, Los Angeles, both in United States. The patients’ electronic or paper medical records were reviewed and clinical history, laboratory test results, imaging studies, and pathological reports were analyzed and extracted. This study has been approved by the Institutional Review Board of the respective participating centers.

### Diagnosis and Treatment

The diagnosis and management approaches were dictated by the local standards but were mostly similar.^11–14^ Briefly, diagnosis of PNETs was suspected if the patients exhibited a hormonal excess syndrome and imaging identified pancreatic or liver masses, or the patients had pancreatic or liver masses either incidentally discovered or found during the workup of abdominal symptoms. In either case, biopsy of the pancreatic mass or liver lesions was done to establish the diagnosis of PNETs. For treatment, the pancreatic PNET and liver metastatic lesions were resected whenever possible, followed by locoregional therapy of residual liver lesions and systemic therapy composed of a somatostatin analog and other medications. Additional therapy was dictated by the treating physicians and by availability.

### Pathological Classification

Tumor grading and staging were performed based on the ENETS criteria.^9,10^ For grading, both mitotic index and Ki-67 labeling were measured; when the results were discordant, the higher grade assigned by either was defined as the grade of the tumor. For staging, the TNM system was used.

### Statistical Analysis

Student *t* test, χ-test or Fisher exact test, and Mann–Whitney method were used. Logistic regression was used for multivariate analysis. Survival of patients was analyzed by the Kaplan–Meier method and Cox proportional hazard model. Two-tailed test was used in all statistical analyses. *P* ≤ 0.05 was considered significant.

## RESULTS

### General Description of PNETs

#### PNET Presentation

Four of the 7 Chinese centers locate in Northern part of China, and 3 in Southern China, respectively. The 2 US centers locate in the East and West Coast of the United States, respectively. The 977 patients’ clinical information is summarized in Tables 1 and 2. Their median age was 51 but the Chinese patients were about 10 years younger than the US patients. Slightly more patients were female (55.5%), especially in China (59.0% vs 51.4% in United States, *P* = 0.0196). Cumulatively, more patients harbored nonfunctional tumors (NF) than functional ones but most of the patients with NF were from US centers. Among the 527 Chinese patients, only 209 had NF (39.7%), while among the 450 US patients, 345 did (76.7%). Of the functional tumors, insulinomas were the most common in both countries, followed by gastrinomas and glucagonomas, and insulinomas were the predominant tumor type in Chinese patients (52.2%). In both countries, the vast majority of patients had sporadic PNETs and ∼5% had familial syndromes such as multiple endocrine neoplasia type 1 (MEN1) and von Hippel-Lindau disease (VHL). Although about 60% of all the primary tumors located at the body or tail while the remainder at the head or neck of pancreas, the Chinese PNETs had an even distribution (49.6% vs 49.0%, Table 1) and 2/3 of US PNETs were at the body/tail region. At presentation, 32.4% of patients already had metastasis but the Chinese patients had less metastasis than US counterparts (28.0% vs 38.4%, *P* = 0.0013, Table 1), mostly due to that the Chinese had larger number of insulinomas that do not usually metastasize. Most patients underwent surgical resection of the primary tumor or liver metastatic lesions but <5% did not due to extensive tumor burden. The most common site of distant metastases was liver, followed by bone, lung, and kidney. One Chinese patient with NF exhibited metastasis to scalp skin.

**TABLE 1:**
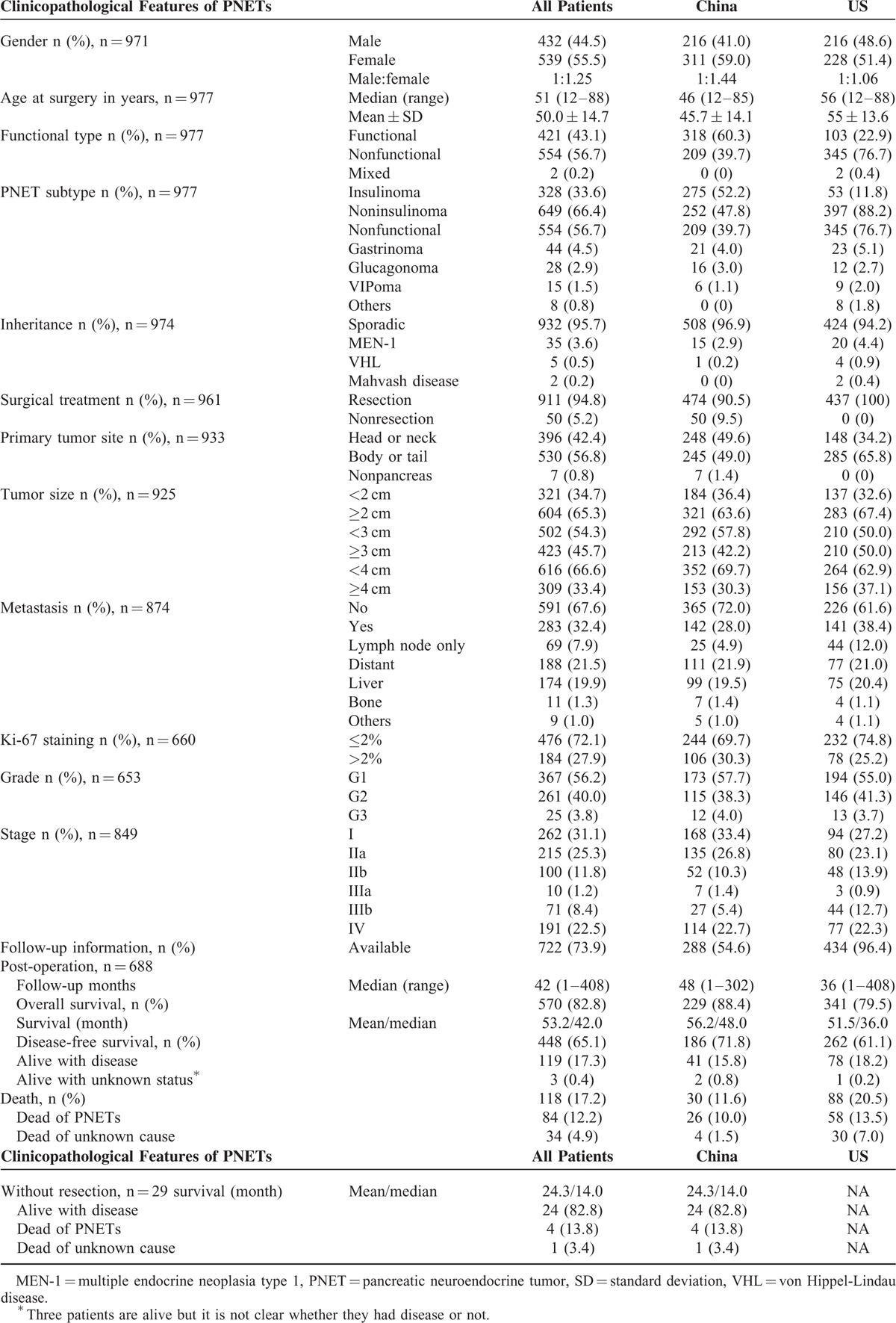
Summary of Clinicopathological Characteristics of 977 Patients With Pancreatic Neuroendocrine Tumors

**TABLE 2:**
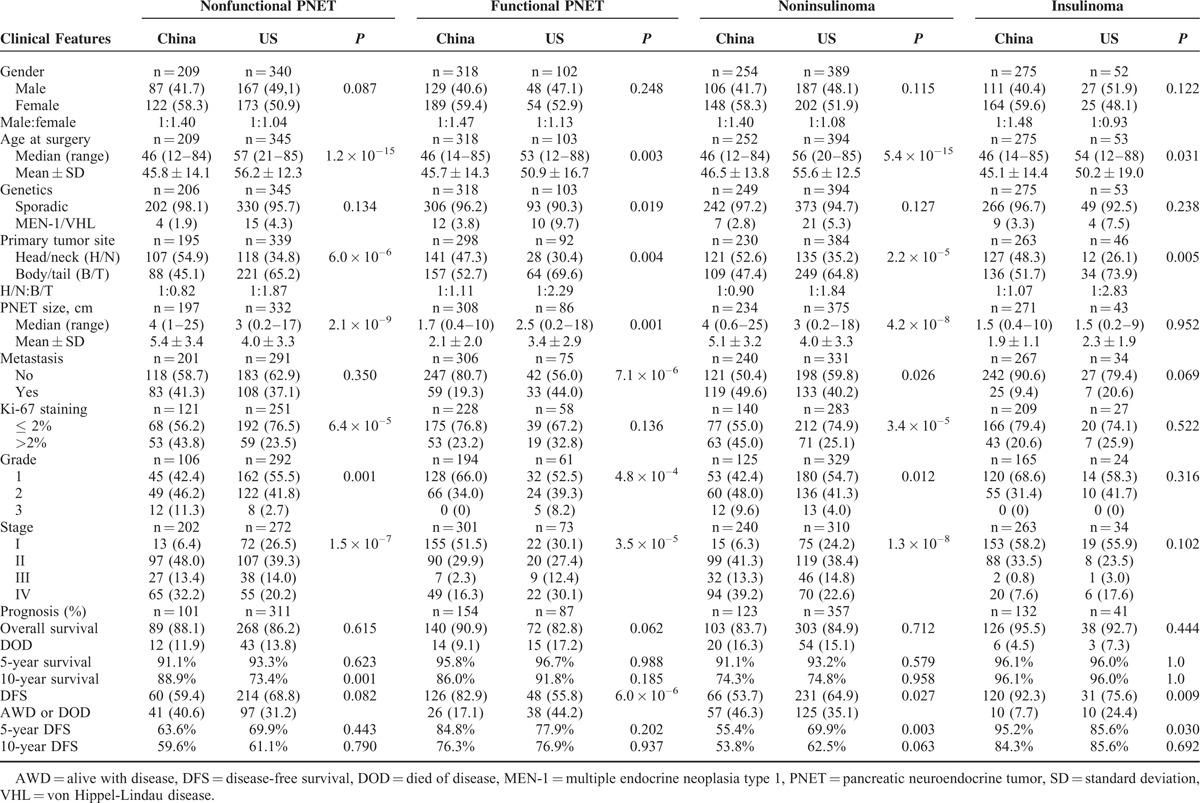
Comparison of Clinicopathological Features of Subtypes of Pancreatic Neuroendocrine Tumors in Chinese and US Patients

#### PNET Grade and Stage

Overall 72.1% of the PNETs had a Ki-67 labeling index ≤2% and there was a small difference in the percentage of tumors with Ki-67 labeling index ≤2% in Chinese and US patients (69.7% vs 74.8%). As mitotic index was also used to grade PNETs, tumors with G1 grade were fewer than those with a Ki-67 labeling index ≤2%. The Chinese and US patients had similar distribution of G1 (57.7% vs 55.0%), G2 (38.3% vs 41.3%), and G3 (4.0 vs 3.7%) PNETs (Table 1). At presentation, most tumors were at stage I, IIa, or IV in both Chinese and US patients, and the Chinese patients had more tumors at stage I or IIa than US patients (61.2% vs 50.3%, *P* = 0.0074, Table 1).

### Analysis of PNET Prognosis

**FIGURE 1:**
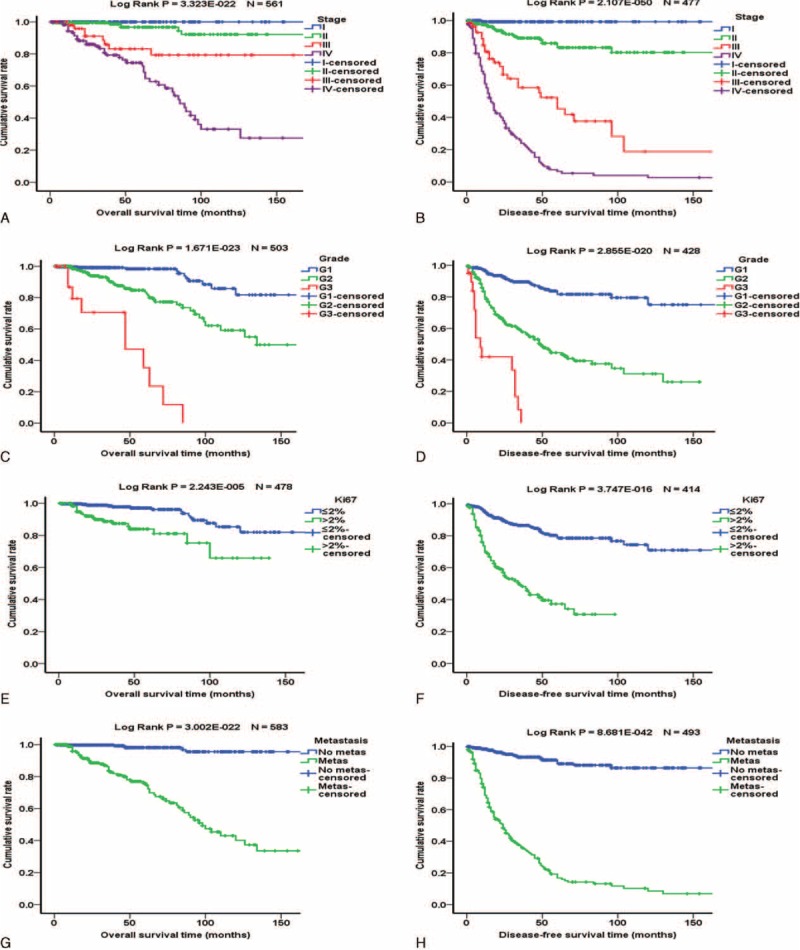
Kaplan–Meier analysis of overall survival and disease-free survival of patients with PNETs. Left panels are the overall survival curves and right panels are disease-free survival curves. (A and B) Influence of tumor stage. Blue, green, red, and purples lines represent patients with stages I, II, III, and IV PNETs, respectively. (C and D) Influence of tumor grade. Blue, green, and red lines represent patients with G1, G2, and G3 PNETs, respectively. (E and F) Influence of Ki-67 labeling index. Blue and green lines represent patients with PNETs with Ki-67 labeling index ≤2% and >2%, respectively. (G and H) Influence of metastasis. Blue and green lines represent patients without and with metastasis, respectively.

**FIGURE 2:**
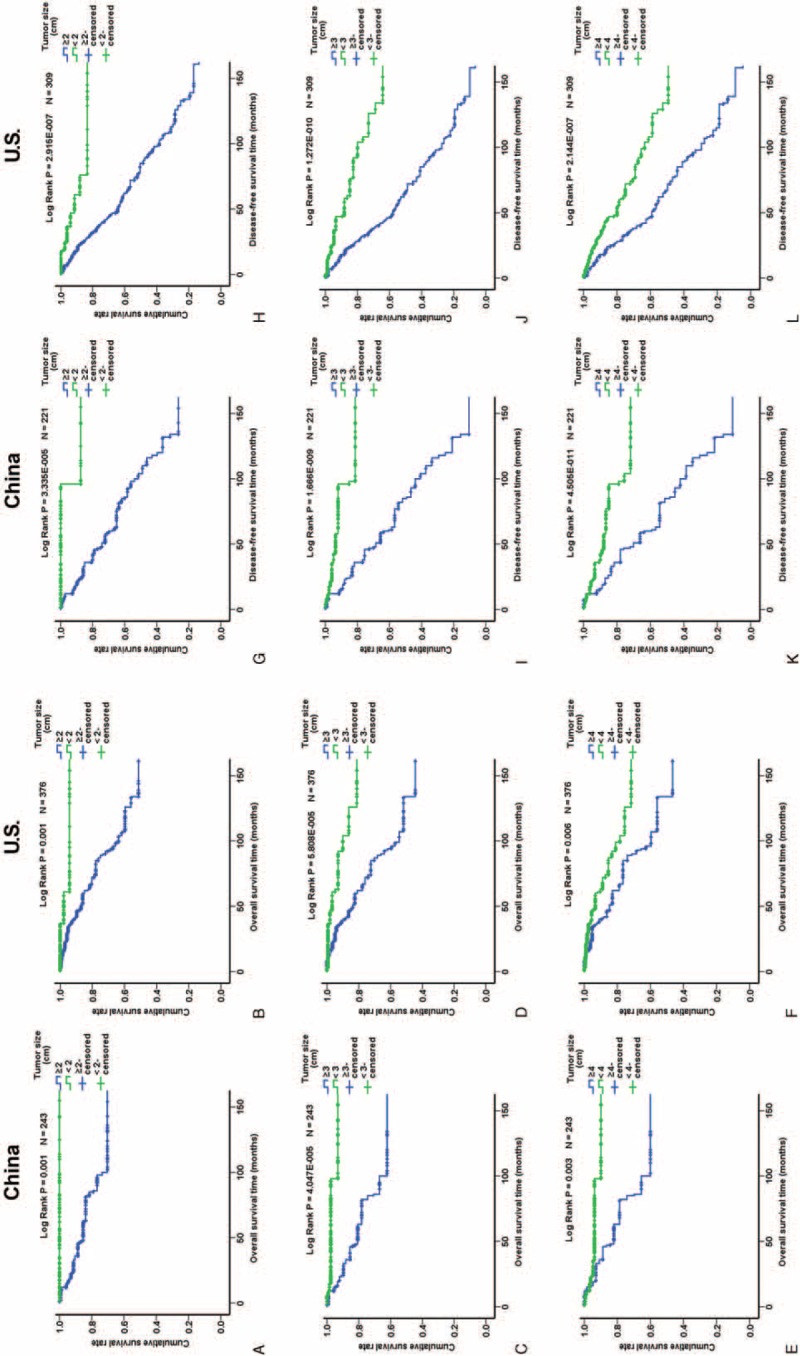
Kaplan–Meier analysis of overall survival and disease-free survival of Chinese and US patients in relation to tumor size. (A–F) Overall survival; (G–L) disease-free survival. Green and blue lines represent patients with tumors <2 and ≥2 cm, respectively (A, B, G, and H), <3 and ≥3 cm, respectively (C, D, I, and J), and <4 and ≥4 cm, respectively (E, F, K, and L).

**FIGURE 3:**
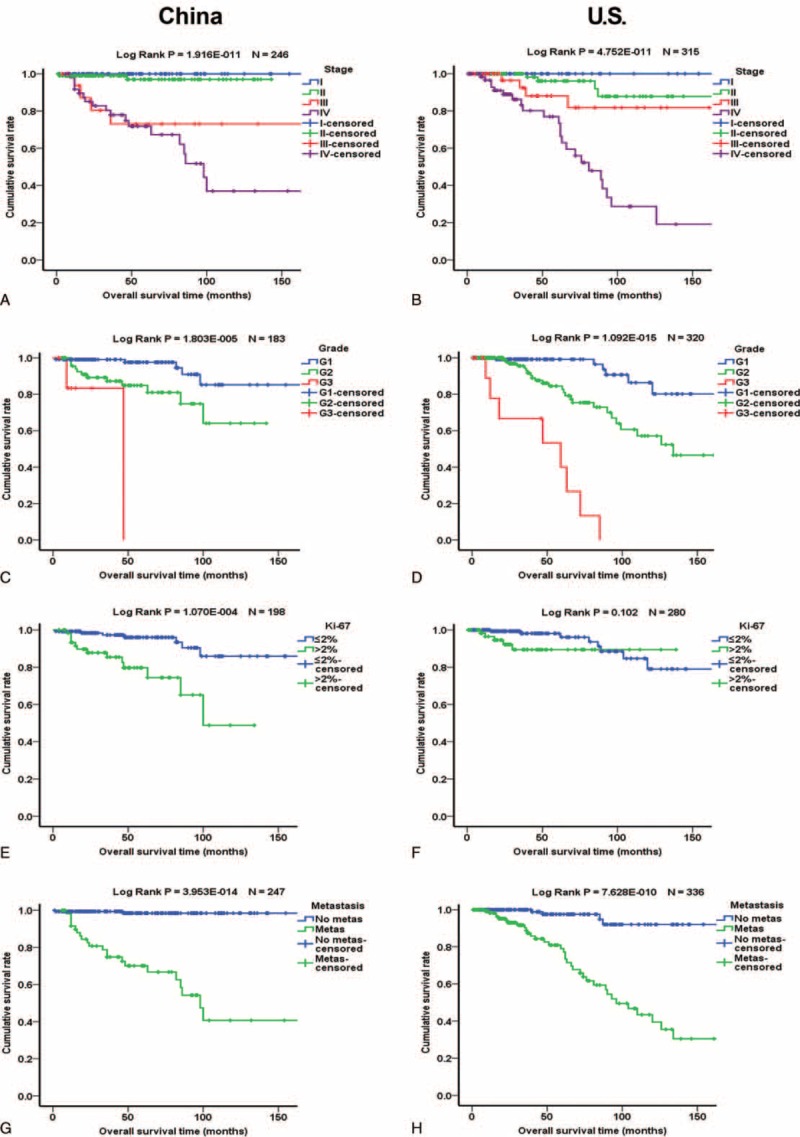
Comparison of Kaplan–Meier analyses of overall survival of Chinese and US patients. Left panels are the curves of Chinese and right panels of US patients. (A and B) Influence of tumor stage. Blue, green, red, and purples lines represent patients with stage I, II, III, and IV PNETs, respectively. (C and D) Influence of tumor grade. Blue, green, and red lines represent patients with G1, G2, and G3 PNETs, respectively. (E and F) Influence of Ki-67 labeling index. Blue and green lines represent patients with PNETs with Ki-67 labeling index ≤2% and >2%, respectively. (G and H) Influence of metastasis. Blue and green lines represent patients without and with metastasis, respectively.

**FIGURE 4:**
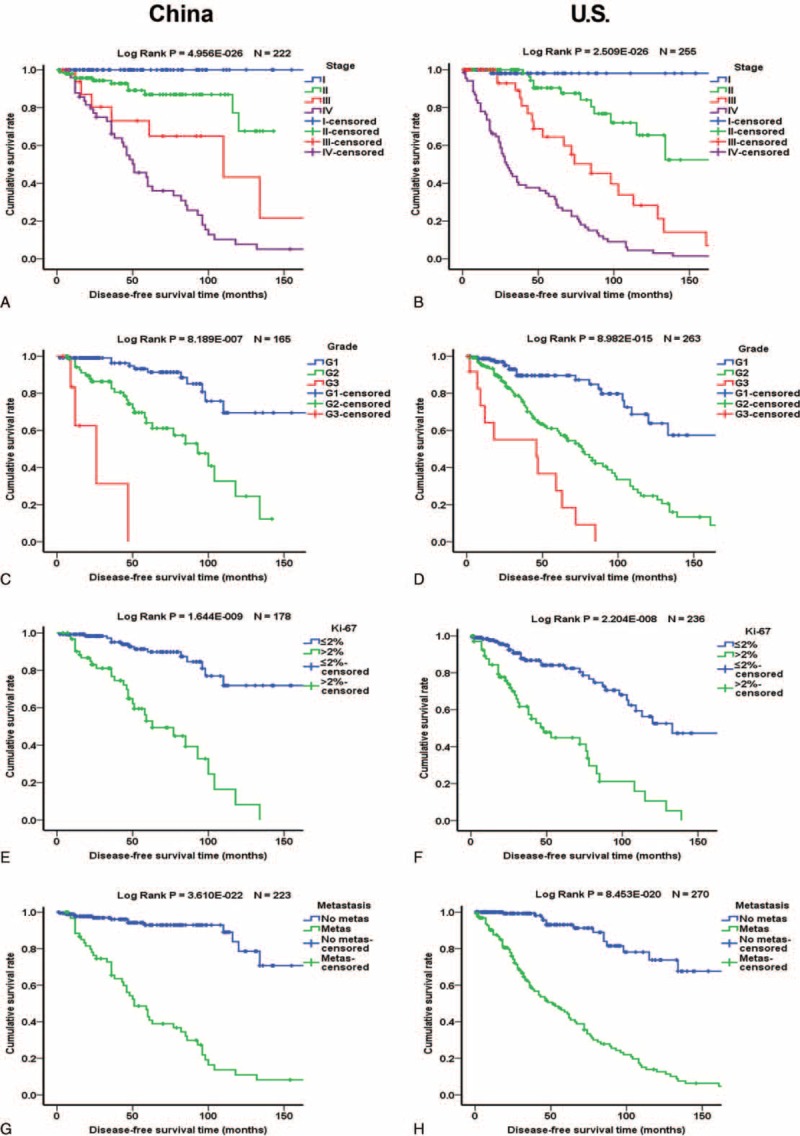
Comparison of Kaplan–Meier analyses of disease-free survival of Chinese and US patients. Left panels are the curves of Chinese and right panels of US patients. (A and B) Influence of tumor stage. Blue, green, red, and purples lines represent patients with stage I, II, III, and IV PNETs, respectively. (C and D) Influence of tumor grade. Blue, green, and red lines represent patients with G1, G2, and G3 PNETs, respectively. (E and F) Influence of Ki-67 labeling index. Blue and green lines represent patients with PNETs with Ki-67 labeling index ≤2% and >2%, respectively. (G and H) Influence of metastasis. Blue and green lines represent patients without and with metastasis, respectively.

Of the 977 patients, 722 (73.9%) had follow-up data, and 688 of the 722 patients underwent surgical resection. The median follow-up period was 42 months. Kaplan–Meier analysis showed that stage, grade, Ki-67 labeling index, and metastasis were significantly associated with overall survival (OS) and disease-free survival (DFS) (Figure 1). Female patients had slightly better OS but the difference was only a statistical trend (Suppl. Figure 1). Functional PNETs exhibited better OS and DFS which was mainly influenced by the excellent prognosis of insulinomas (Suppl. Figure 1). Larger primary tumor size was significantly associated with worse prognosis in both Chinese patients and US patients whatever cutoff value, 2-, 3-, or 4 cm, was used (Figure 2 and Suppl. Figure 2). Separate Kaplan–Meier analyses of Chinese and US patients did not reveal significant differences in the relationships between age, sex, functionality, metastasis, Ki-67 labeling index, grade, and stage and OS (Figure 3 and Suppl. Figure 3) or DFS (Figure 4 and Suppl. Figure 4), except for that in US patients, Ki-67 labeling index >2% was not significantly associated with worse OS (*P* = 0.102, Figure 3F).

Multivariate analysis (Cox models) suggested that higher grades, more advanced stages and presence of metastasis were associated with unfavorable OS or DFS (Suppl. Tables 1 and 2). Primary tumor size <3 cm was significantly associated with favorable DFS and a statistical trend of better OS (Suppl. Table 1). When we correlated the primary tumor size with OS in Chinese and US cohorts separately, the tumor size was not associated with OS as the number of patients was smaller in each group (Suppl. Table 2). The associations of the above variables with survival were generally similar in Chinese and US patients (Suppl. Table 2).

### Comparison of Clinicopathological Features Between PNET Subgroups of Chinese and US Patients

As one of the aims of the study was to compare the clinicopathological characteristics between Chinese and US PNETs, we further compared different subtypes of Chinese and US PNETs. Age, tumor size, primary location, grade, and stage were significantly different between Chinese and US patients within the same subgroups of PNETs. These data are summarized in Table 2.

#### Nonfunctional PNETs (NF)

When the Chinese and US patients were considered as a single group, a total of 554 patients had NF with more from United States (n = 345, 62.3%) (Tables 1 and 2). The Chinese NF were significantly larger than United States ones (median (range) 4 (1–25) vs 3 (0.2–17) cm; mean ± SD 5.4 ± 3.4 vs 4.0 ± 3.3 cm) and more frequently located in the head/neck region (54.9% vs 34.8%). The Chinese patients had lower percentage of G1 (42.4% vs 55.5%) and higher G3 (11.3% vs 2.7%) tumors than US patients (*P* = 0.001). The Chinese patients also had lower percentage of stage I (6.4% vs 26.5%) and higher percentage of stage IV (32.2% vs 20.2%) tumors than US patients (*P* = 1.5 × 10^–7^) (Table 2). The rates of 5-year OS of Chinese and US patients were similar (91.1% vs 93.3%) whereas the rate of 10-year OS of Chinese patients was modestly but significantly higher (88.9% vs 73.4%). The rates of DFS were similar in the Chinese and US patients: 63.6% vs 66.9% at 5 years, and 59.6% vs 61.1% at 10 years (Table 2).

#### Insulinomas and Noninsulinoma PNETs

As insulinomas are the most common functional PNETs, they were analyzed as a single group composed of patients from both countries or separately. A total of 328 patients had insulinoma with most of them from China (n = 275, 83.8%). Noninsulinoma PNETs were the largest subgroup of PNETs, including 649 patients with 61.2% of them from the United States. The median size of insulinomas was the same in Chinese and US patients (1.5 vs 1.5 cm), in contrast, the median size of noninsulinoma tumors was 4.0 and 3.0 cm in Chinese and US patients, respectively (*P* = 4.2 × 10^–8^, Table 2). The Chinese insulinomas relatively more frequently located in the head/neck region (48.3% vs 26.1%). The Chinese and US insulinomas had similar distribution of G1 (68.6% vs 58.3%), G2 (31.4% vs 41.7%), and G3 (0% vs 0%) whereas the distribution of grade was quite different in the noninsulinomas between 2 countries (*P* = 0.012). In both countries, most insulinomas were at stages I and II. The Chinese insulinoma patients had a trend of lower percentage of stages III and IV combined than US patients (8.4% vs 20.6%, *P* = 0.102) but this was reversed in patients with noninsulinomas (52.5% vs 37.4%, *P* = 1.3 × 10^–8^, Table 2). The rate of 5- and 10-year OS of Chinese and US insulinoma patients were similar (96.1% vs 96.0%). The rate of 5-year DFS of the Chinese insulinoma patients was slightly higher than that of US patients (95.2 % vs 85.6%, *P* = 0.030) but the rates of 10-year DFS were similar (84.3% vs 85.6%) (Table 2). In contrast, the rate of 5-year DFS of the Chinese noninsulinoma patients was significantly lower than that of US patients (55.4 % vs 69.9%, *P* = 0.003).

## DISCUSSION

In this study, we compared the presentation, pathology, and prognosis of PNETs in Chinese and US patients. To our knowledge, this is the first multicenter, systemic study of PNETs in a large cohort of Chinese patients. The use of US patients as control has allowed us to validate the results of the study. We demonstrate interesting differences in the presentation of PNETs and gross similarities in the pathology and prognosis of PNETs among Chinese and US patients.

It may be initially surprising that most Chinese patients had functional while most US patients had nonfunctional PNET (NF) but evolution of the epidemiology of NF in the United States suggests that the smaller NF case numbers in Chinese patients may be due to a detection bias. NF used to be considered rare in US populations as well but has become more common than functional tumors in recent years. At Mayo Clinic, the percentage of NF increases from 15% between 1960 and 1978 to 53% between 1985 and 1993.^17,18^ Similarly, at MD Anderson Cancer Center, the percentage of NF increases from 44% between 1950 and 1987 to 91% between 2001 and 2009.^19,20^ The same increase of the frequency of NF is also observed at Massachusetts General Hospital.^21^ The increase of the frequency of NF is most likely due to early detection of NF by sensitive imaging modalities such as CT and MRI before the tumor causes clear symptoms. In China where sophisticated imaging is currently probably not as widely used as in the United States,^22,23^ early detection of NF is thus less likely, explaining its apparent lower relative incidence than that of functional tumors. On the other hand, we cannot rule out that NF is genuinely uncommon in China. Future epidemiological studies of PNETs in Chinese patients would clarify this issue.

Although most Chinese patients with PNETs were middle-aged, like the US patients, the apparent younger age of presentation of insulinomas and NF in Chinese patients is intriguing. As there are no known environmental or behavioral factors contributing to PNETs pathogenesis,^1–5^ it is presumptive to discuss factors that make Chinese PNET patients present at younger ages. However, there could be several potential underlying causes of this phenomenon. The Chinese patients may be genetically more susceptible to PNETs, thus presenting at a younger age. Since more Chinese NFs locate in pancreatic head or neck where the tumors tend to cause symptoms earlier than tumors at pancreatic body or tail, Chinese NF patients may thus present at an earlier age. Moreover, the tumor size of NF in Chinese patients was relatively larger than that of US patients, which also results in symptoms at earlier stage of the tumor. Another possible explanation of the younger age of Chinese patients with PNETs is that the life expectancy of Chinese people (74.8 years) is about 3 years shorter than that of US people (78.2 years), so that older Chinese patients who would otherwise be diagnosed with PNETs may die of other causes before being diagnosed. Interestingly, the age of patients with PNETs in the United States is getting older since 1960s. For example, at MD Anderson Cancer Center, the mean age of patients with PNETs used to be younger as well but grew from 49 years between 1950 and 1987 to 58 years between 2001 and 2009.^19,20^ The higher female to male ratio in Chinese PNET patients (1.44:1) is different from the equal sex distribution in US patients but similar to the sex ratio (female to male: 1.6:1) in Japanese patients.^6^ These findings suggested that PNETs in Eastern Asian countries might be different from those in the United States and further studies are needed to address the underlying causes of the different sex ratio.

The larger size of the Chinese NF is also interesting. The Chinese NF may be biologically prone to cell proliferation or to allow more rapid tumor expansion. This hypothesis is supported by our present finding that almost twice as much Chinese NF had Ki-67 >2%, compared with US NF (43.8% vs 23.5%). In addition, there might be unknown environmental factors present in China to promote PNET growth. Alternatively, the size difference may be a result of symptom-based tumor detection. As small NFs are usually asymptomatic, especially when they are located in the body/tail of pancreas, they will remain undetected until incidentally identified by imaging.^1–5^ The symptom-based diagnostic workup thus mostly identifies larger tumors and tumors at the head/neck of pancreas, which are more common in the Chinese patients. Before the advent of frequent imaging procedures in the United States, NFs were also larger than those seen in contemporary practice. At Massachusetts General Hospital, for example, the average size of NFs decreases from 5.6 cm between 1977 and 1999 to 4.1 cm between 2000 and 2005.^21^

Despite the several differences in the presentation of PNETs in Chinese and US patients, pathological classification of PNETs by the same grading and staging criteria appears to be feasible and validated. The Chinese NFs that tend to be larger (thus suggesting higher proliferation rate) are indeed classified into higher grades and more advanced stages while the Chinese insulinomas which are very similar to the US counterparts are appropriately classified into lower grades and earlier stages. Tumor grade distribution shown in our study is similar between Chinese and US patients, and is close to those from previous larger studies in Europe and United States, especially a large multicenter European study,^10,24–27^ and a small study in Japan,^16^ suggesting that in academic centers, most PNETs present as G1, followed by G2, and remotely by G3 grade. Survival data stratified by the WHO/ENETS grades are also consistent with data from validation studies in European and other US centers,^24–27^ supporting the validity of WHO/ENETS grading in Asian countries and for Asian patients. Likewise, the distribution of tumor stage in our study is also approximately similar between Chinese and US patients, and the distributions of Stages I and IV tumors are very similar to those reported in a large multicenter study.^10^ In addition, the survival data stratified by ENETS TNM staging are similar between Chinese and US patients, and are largely close to those from previous studies using similar or other TNM-based staging systems.^25,26,28–30^ Our study thus indicates that the ENETS grading and staging criteria can be universally applied to PNETs in most countries.

The mostly similar prognosis of PNETs in Chinese and US patients suggests that the prognosis of PNETs is largely a function of tumor grade and stage, so long as surgical resection of primary and metastatic tumors and locoregional therapy of liver metastatic tumor, the 2 common treatments invariably used whenever feasible in both countries. It has been suggested that somatostatin analogs may improve the survival of patients with small bowel carcinoids and PNETs.^31^ Although the 7 Chinese centers are among the best academic hospitals in China, the use of somatostatin analogs and other medications is often limited by patients’ inability to afford them, thus much fewer Chinese patients received these medications but unfortunately the accurate data are not available to us. Our study thus cannot reliably address the survival benefits of somatostatin analogs.

Our study has several limitations. As described earlier, we did not have data on detailed systemic treatment on most Chinese patients. This is a retrospective study with the intrinsic shortcomings of a retrospective study. The Chinese and US patient populations contain uneven proportions of functional tumors and NF, making the comparison of overall PNET prognosis less feasible. We used survival, a more definitive measure of prognosis, but did not have data on patients’ symptoms and quality of life, which are both important measures of prognosis. These limitations, however, do not diminish the value of our main findings.

In summary, this multicenter, cross-Pacific study of nearly 1000 patients with PNETs demonstrates that the contemporary Chinese patients in general are younger, have relatively fewer nonfunctional tumors, and larger tumors than US patients. Tumor grades and stages are also different in subgroups of PNETs between the 2 countries. PNET prognosis is similarly associated with tumor grade, stage, metastasis, and primary tumor size. Thus, PNETs in Chinese and US patients likely follow a similar natural history and the ENETS grading and staging criteria are validated in Chinese patients. The same criteria can probably be used in Asian patients at large.

## Supplementary Material

Supplemental Digital Content
