# Supplementary material for: Differences and Similarities in the Clinicopathological Features of Pancreatic Neuroendocrine Tumors in China and the United States: A Multicenter Study
Source: Medicine (Baltimore). 2016 Feb 18;95(7):e2836. doi: 10.1097/MD.0000000000002836 (PMC4998644; doi:10.1097/MD.0000000000002836)

**Supplemental Table 1. Multivariable Cox Models for Factors Associated with Overall Survival and Disease-Free Survival of All Patients with follow-up data**

| Model variables | Overall survival | | Disease-free survival | |
| --- | --- | --- | --- | --- |
|  | HR (95% CI) | *P* | HR (95% CI) | *P* |
| Model 1 |  |  |  |  |
| Age | 0.997 (0.971 to 1.024) | 0.815 | 0.988 (0.969 to 1.008) | 0.253 |
| Gender | 0.722 (0.399 to 1.307) | 0.282 | 0.982 (0.574 to 1.680) | 0.947 |
| Grade | 4.201 (2.235 to 7.894) | 8.25 E-06 | 3.337 (1.885 to 5.908) | 3.54 E-05 |
| Stage | 3.034 (2.048 to 4.495) | 3.12 E-08 | 3.518 (2.595 to 4.769) | 5.37 E-16 |
| NF/functional PNETs | 1.571 (0.751 to 3.285) | 0.230 | 0.665 (0.360 to 1.229) | 0.193 |
| Non-insulinomas/ Insulinomas | 0.466 (0.136 to 1.599) | 0.225 | 1.994 (0.769 to 5.171) | 0.156 |
| Model 2 |  |  |  |  |
| Age | 0.999 (0.975 to 1.023) | 0.910 | 0.985 (0.967 to 1.004) | 0.119 |
| Size <3 cm | 0.457 (0.196 to 1.064) | 0.069 | 0.344 (0.155 to 0.764) | 0.009 |
| Metastasis | 9.887 (3.911 to 24.995) | 1.28 E-06 | 8.432 (4.337 to 16.394) | 3.27 E-10 |
| Grade | 4.311 (2.310 to 8.048) | 4.46 E-06 | 3.438 (1.965 to 6.015) | 1.51 E-05 |
| NF/functional PNETs | 1.147 (0.573 to 2.297) | 0.698 | 0.579 (0.316 to 1.060) | 0.077 |
| Non-insulinomas/  Insulinomas | 0.457 (0.134 to 1.554) | 0.210 | 1.450 (0.520 to 4.041) | 0.478 |
| Model 3 |  |  |  |  |
| Age | 0.990 (0.962 to 1.018) | 0.468 | 0.975 (0.955 to 0.995) | 0.013 |
| Size <3 cm | 0.372 (0.110 to 1.258) | 0.112 | 0.217 (0.082 to 0.576) | 0.002 |
| Metastasis | 17.069 (4.819 to 60.456) | 1.10 E-05 | 10.566 (4.745 to 23.528) | 7.83 E-09 |
| Ki-67 >2% | 2.034 (0.927 to 4.459) | 0.076 | 3.455 (1.841 to 6.485) | 1.14 E-04 |
| NF/functional PNETs | 1.643 (0.660 to 4.092) | 0.286 | 0.791 (0.400 to 1.565) | 0.501 |
| Non-insulinomas/ Insulinomas | 0.391 (0.103 to 1.492) | 0.170 | 1.016 (0.335 to 3.076) | 0.978 |

NF: non-functional

**Supplemental Table 2. Multivariable Cox Models for Factors Associated with Overall Survival and Disease-Free Survival of Chinese and US patients, analysed separately.**

| Model variables | Chinese PNETs | | | | American PNETs | | | |
| --- | --- | --- | --- | --- | --- | --- | --- | --- |
|  | Overall survival | | Disease-free survival | | Overall survival | | Disease-free survival | |
|  | HR (95% CI) | *P* | HR (95% CI) | *P* | HR (95% CI) | *P* | HR (95% CI) | *P* |
| Model 1 |  |  |  |  |  |  |  |  |
| Age | 1.006 (0.970 to 1.044) | 0.744 | 0.993 (0.965 to 1.021) | 0.621 | 0.973 (0.937 to 1.011) | 0.160 | 0.989 (0.962 to 1.017) | 0.441 |
| Gender | 1.468 (0.568 to 3.795) | 0.429 | 1.569 (0.677 to 3.632) | 0.293 | 0.417 (0.180 to 0.970) | 0.042 | 0.899 (0.411 to 1.966) | 0.789 |
| Grade | 2.560 (1.027 to 6.380) | 0.044 | 2.969 (1.332 to 6.616) | 0.008 | 5.655 (2.403 to 13.311) | 7.28 E-06 | 4.240 (1.716 to 10.481) | 0.002 |
| Stage | 3.004 (1.712 to 5.270) | 1.26 E-04 | 4.585 (2.836 to 7.413) | 5.19 E-10 | 2.841 (1.665 to 4.848) | 1.28 E-04 | 3.437 (2.281 to 5.179) | 3.63 E-09 |
| Model 2 |  |  |  |  |  |  |  |  |
| Age | 1.006 (0.968 to 1.046) | 0.744 | 0.986 (0.959 to 1.014) | 0.330 | 0.989 (0.955 to 1.023) | 0.518 | 0.991 (0.966 to 1.017) | 0.495 |
| Size <3 cm | 0.542 (0.126 to 2.337) | 0.411 | 0.309 (0.100 to 0.958) | 0.042 | 0.545 (0.193 to 1.541) | 0.252 | 0.338 (0.111 to 1.025) | 0.055 |
| Metastasis | 15.564 (3.029 to 79.974) | 0.001 | 11.936 (4.290 to 33.208) | 2.04 E-06 | 6.044 (2.081 to 17.554) | 0.001 | 6.977 (3.163 to 15.387) | 1.48 E-06 |
| Grade | 1.991 (0.751 to 5.281) | 0.167 | 2.725 (1.201 to 6.180) | 0.016 | 5.710 (2.610 to 12.491) | 1.29 E-05 | 4.381 (1.934 to 9.922) | 3.99 E-04 |
|  |  |  |  |  |  |  |  |  |
| Model 3 |  |  |  |  |  |  |  |  |
| Age | 1.012 (0.976 to 1.049) | 0.514 | 0.983 (0.956 to 1.010) | 0.216 | 0.936 (0.882 to 0.993) | 0.029 | 0.970 (0.939 to 1.002) | 0.063 |
| Size <3 cm | 0.459 (0.105 to 2.007) | 0.301 | 0.353 (0.109 to 1.138) | 0.081 | 0.375 (0.058 to 2.428) | 0.303 | 0.098 (0.012 to 0.774) | 0.028 |
| Metastasis | 16.554 (3.287 to 83.371) | 0.001 | 12.329 (4.130 to 36.804) | 6.74 E-06 | 12.280 (1.470 to 102.610) | 0.021 | 7.384 (2.422 to 22.506) | 4.38 E-04 |
| Ki-67 >2% | 1.555 (0.550 to 4.395) | 0.405 | 5.373 (1.953 to 14.783) | 0.001 | 1.331 (0.384 to 4.617) | 0.652 | 2.316 (0.973 to 5.514) | 0.058 |

**Legends to Supplemental Figures**

Supplemental Figure 1. Kaplan-Meier analysis of overall survival and disease-free survival of all 977 patients with PNETs. Left panels are the overall survival curves and right panels are disease-free survival curves. I and J, influence of age. Blue, green, red, and purples lines represent patients of age <40, 40-49, 50-59, and ≥60, respectively. K and L, influence of sex. Blue and green lines represent male and female patients, respectively. M and N, influence of tumor functionality. Blue and green lines represent patients with function and non-functional PNETs, respectively. O and P, influence of insulinoma. Blue and green lines represent patients with insulinoma and non-insulinoma, respectively.


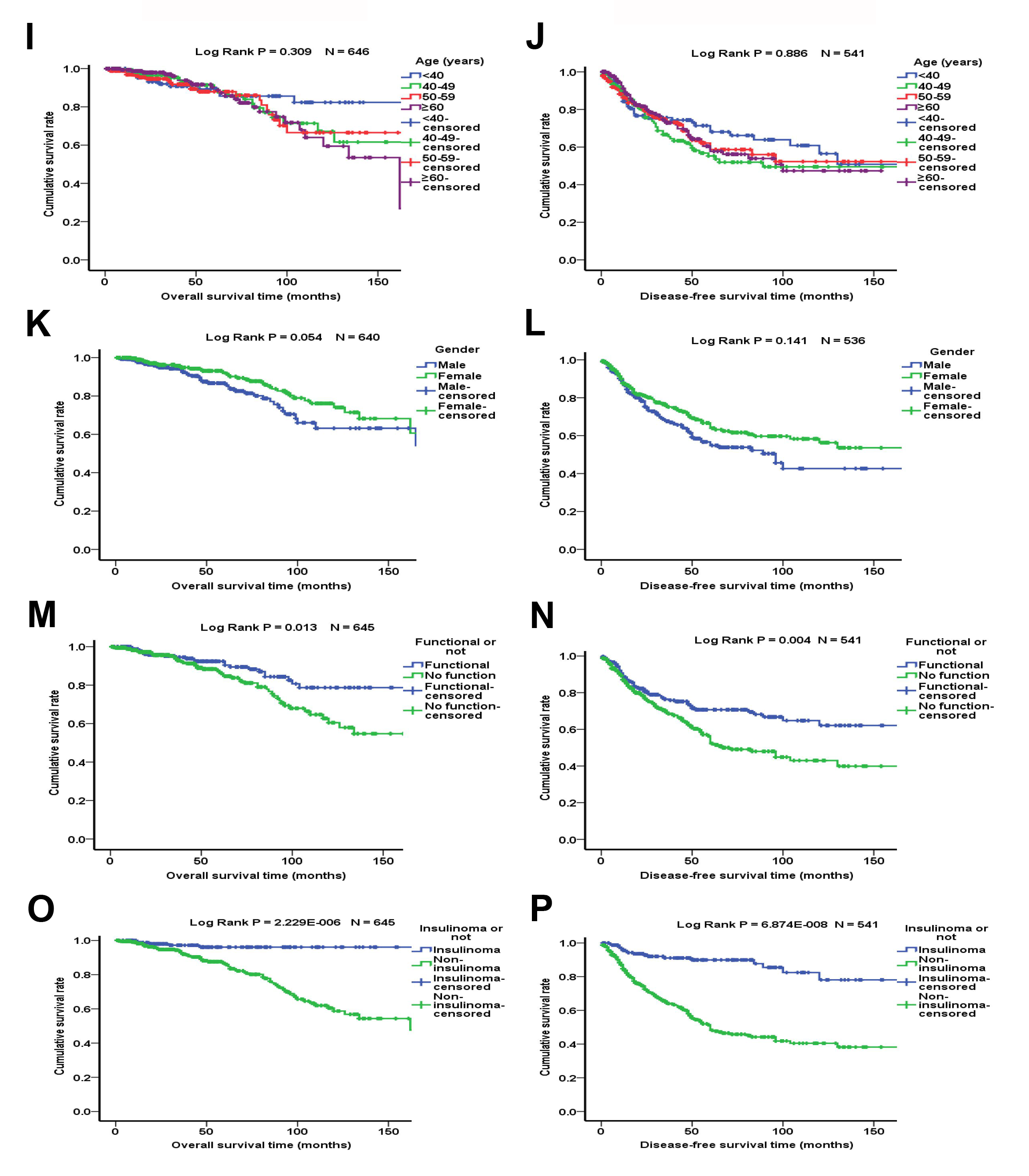


Supplemental Figure 2. Kaplan-Meier analysis of overall survival and disease-free survival of all 977 patients in relation to tumor size. M-O, overall survival; P-R, disease-free survival. Green and blue lines represent patients with tumors <2 cm and ≥2 cm, respectively (M and P), <3 cm and ≥3 cm, respectively (N and Q), and <4 cm and ≥4 cm, respectively (O and R).


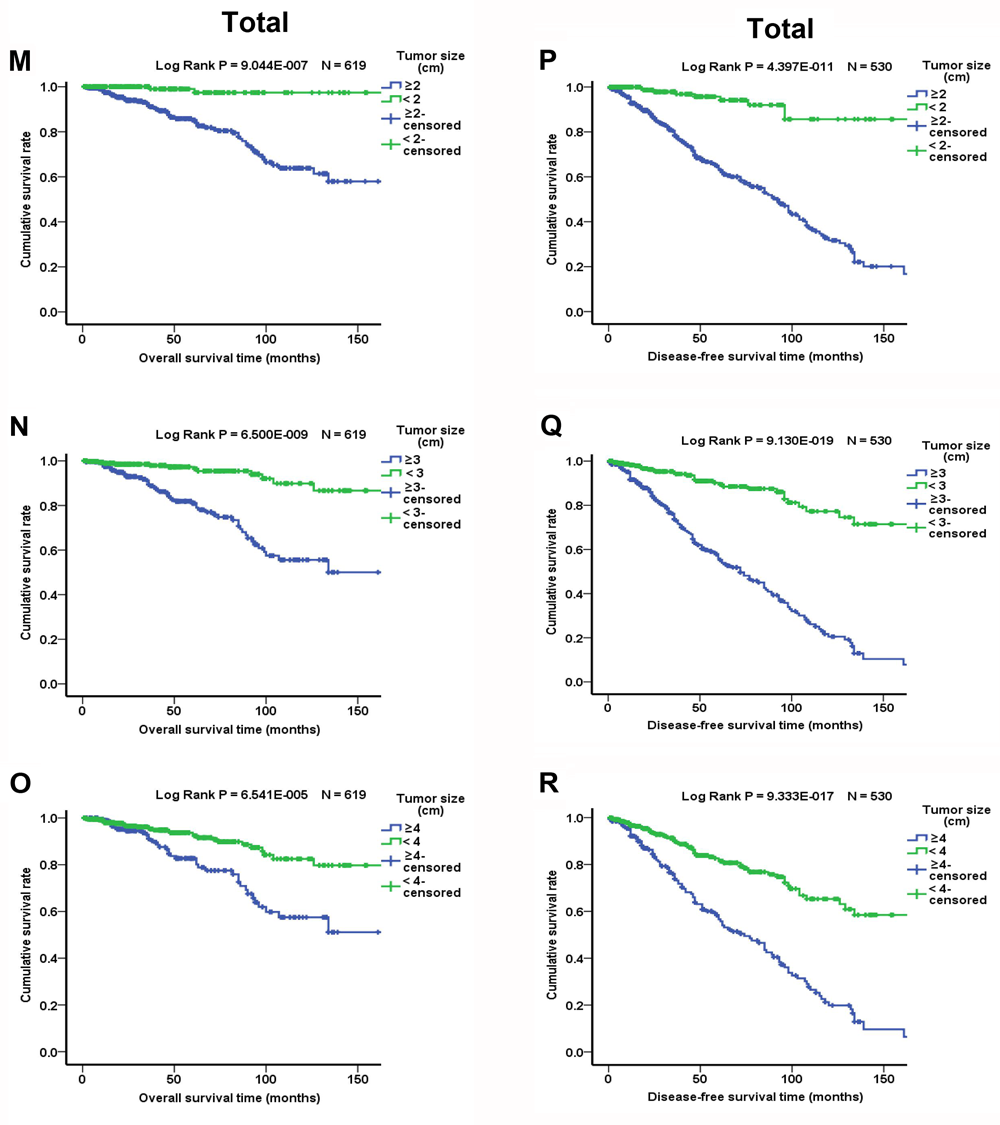


Supplemental Figure 3. Comparison of Kaplan-Meier analysies of overall survival of Chinese and U.S. patients. Left panels are the curves of Chinese and right panels of U.S. patients. I and J, influence of age. Blue, green, red, and purples lines represent patients of age <40, 40-49, 50-59, and ≥60, respectively. K and L, influence of sex. Blue and green lines represent male and female patients, respectively.


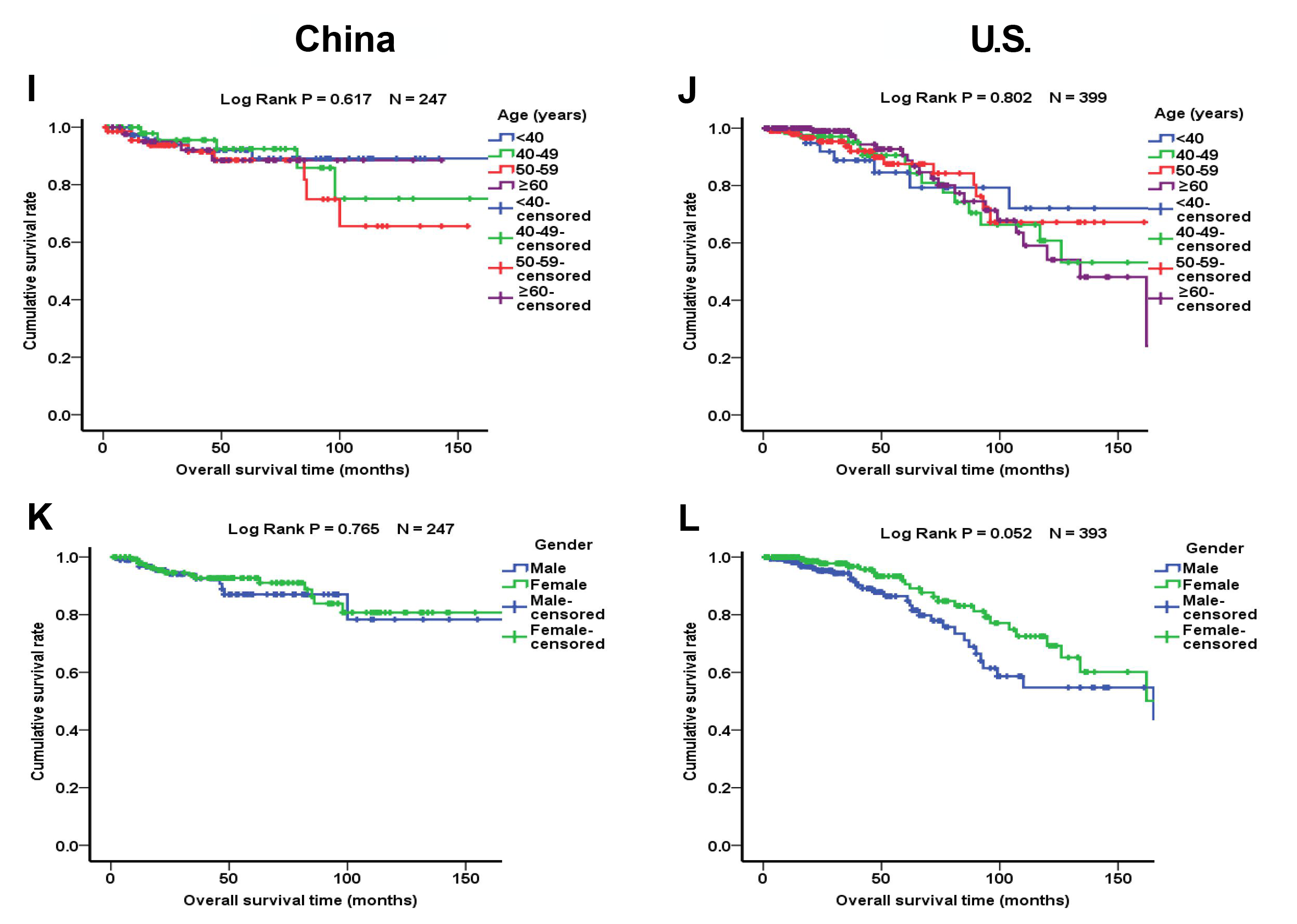


Supplemental Figure 4. Comparison of Kaplan-Meier analyses of disease-free survival of Chinese and U.S. patients. Left panels are the curves of Chinese and right panels of U.S. patients. I and J, influence of age. Blue, green, red, and purples lines represent patients of age <40, 40-49, 50-59, and ≥60, respectively. K and L, influence of sex. Blue and green lines represent male and female patients, respectively.


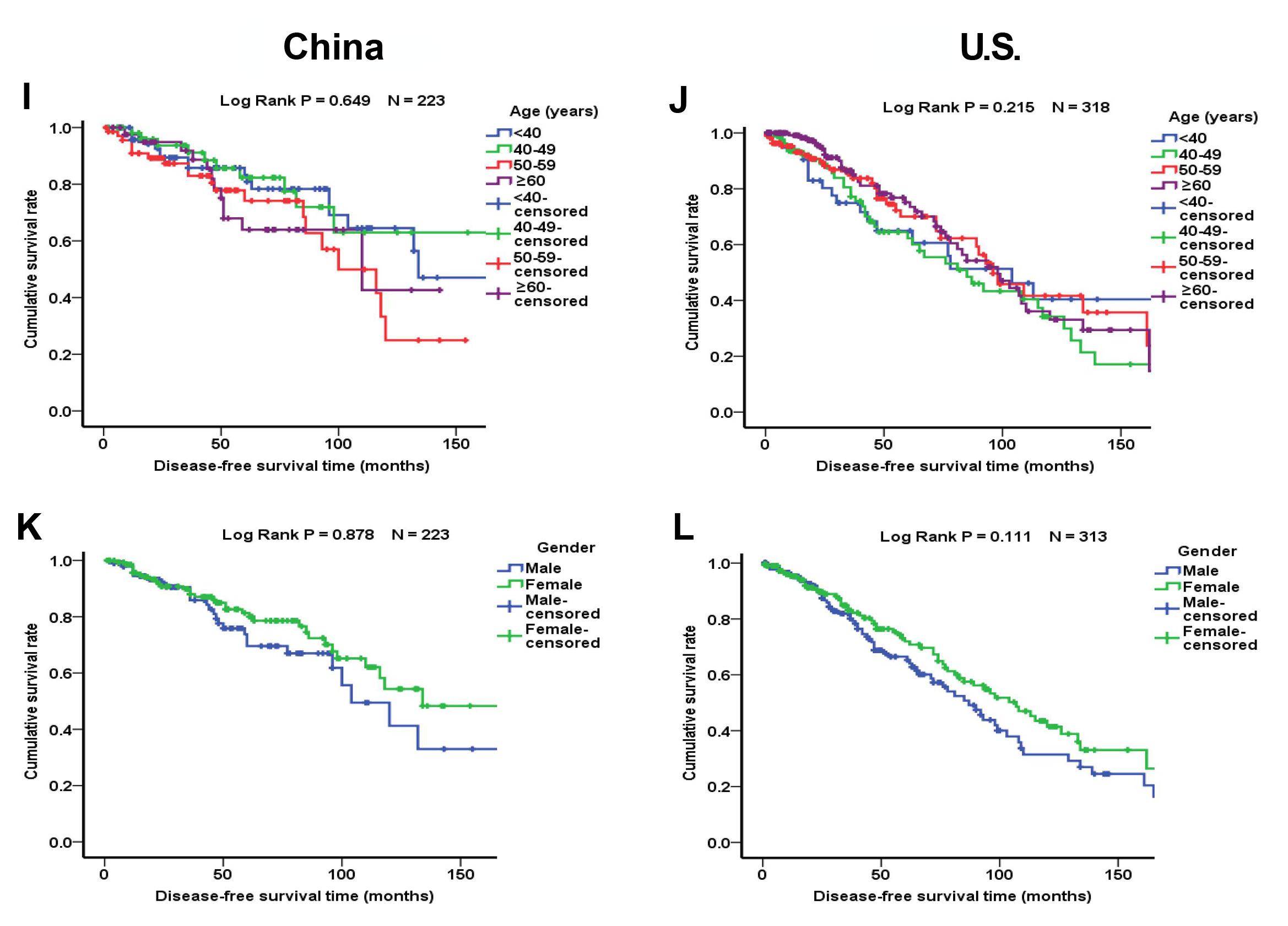

Supplement: Supplemental Digital Content [file medi-95-e2836-s001.doc]
